# Can Cell Bound Complement Activation Products Predict Inherited Complement Deficiency in Systemic Lupus Erythematosus?

**DOI:** 10.1155/2016/8219317

**Published:** 2016-12-15

**Authors:** Naveen Raj, Barry Waters

**Affiliations:** Department of Rheumatology, Nova Southeastern University/Larkin Hospital, Coral Springs, FL, USA

## Abstract

Activation of the classical pathway complement system has long been implicated in stimulating immune complex mediated tissue destruction in systemic lupus erythematosus (SLE). C3 and C4 complement levels are utilized as part of SLE diagnosis and monitoring criteria. Recently, cell bound complement activation products (CBCAPs) have shown increased sensitivity in diagnosing and monitoring lupus activity, compared to traditional markers. CBCAPs are increasingly utilized in rheumatology practice as additional serological markers in evaluating SLE patients. We report a case of a patient diagnosed with SLE that had chronically low C3 and C4, along with negative CBCAPs. We surmise that the patient has an inherited complement deficiency as the etiology of her SLE and that CBCAPs could be used to predict such deficiency.

## 1. Introduction

The pathogenesis of systemic lupus erythematosus (SLE) has long been ascribed to the presence of immune complexes, prompting complement pathway activation and complement consumption. As such, serum complement (i.e., C3 and C4) measurements have been utilized as part of the Systemic Lupus International Collaborating Clinics (SLICC) criteria in diagnosing and monitoring SLE. However, several factors have emerged as evidence of the inadequate utility of serum C3 and C4 in monitoring SLE activity. First, there is a wide range of serum C3 and C4 levels that overlap between healthy individuals and those with SLE. Second, the consumption of C3 and C4 during activation may be counteracted by increased synthesis of complement during active inflammation. Thirdly, hereditary deficiencies in complement such as C4 may result in persistently low complement levels reflective of decreased synthesis rather than increased consumption [1, 2]. The intimate association between complement and SLE and the recognition of serum C3 and C4 as inadequate markers of SLE activity has led investigators to study complement activation products [2].

Over the last decade, measurements of cell bound complement activation products (CBCAPs) have played an increasingly prominent role in aiding in the diagnosis of SLE as well as monitoring disease activity. This is borne out of the rationale that as complement activation occurs during lupus flares, complement is consumed thus lowering its level. Activation derived complement products are then generated at a rate proportional to the degree of disease activity [1, 3]. These complement activation products are stably deposited on various cell membranes including erythrocytes (E C4d) and B-lymphocytes (B C4d) [4]. Erythrocytes, being the most abundant cell in circulation, are particularly prone to accumulating CBCAPs on their cell membranes, which can then be measured by flow cytometry [1]. In 2004, Manzi et al. found that erythrocytes from SLE patients had significantly higher levels of EC-4d when compared to healthy controls or patients with other diseases [5, 6]. In another study of 304 SLE patients, Putterman et al. reported that CBCAPs on erythrocytes or B cells had higher sensitivity than standard complement levels (serum C3 and C4) and anti-dsDNA measurements when distinguishing between SLE and non-SLE [4]. Kao et al. conducted a large prospective trial measuring EC3d and EC4d in 157 SLE patients, 290 patients with other rheumatic diseases, and 256 healthy individuals. The results showed that, at baseline, SLE patients had higher median levels of EC3d and EC4d (*p* < 0.0001) compared to the two non-SLE groups. The results of the study indicated that EC3d and EC4d were informative measures of complement activation and lupus disease activity [2]. The plausible suggestions of these and other studies are that CBCAP is a more specific and sensitive biomarker for the diagnosis and prognosis of SLE compared to serum C3 and C4 [4, 6]. CBCAP measurement has become more widely available in clinical practice and is increasingly being utilized as an additional serological marker, along with the standard SLE measures of anti-nuclear antibody (ANA), anti-Smith, anti-dsDNA, and so forth. To this end, an interesting observation was made in a SLE patient seen in our clinic. She had persistently low serum C3 and C4 levels, measured during an active SLE flare as well as during quiescent periods. Interestingly, her CBCAP levels (EC4d and BC4d) on two separate measurements were negative. This led us to consider whether negative CBCAP in a SLE patient with persistently low serum complement is indicative of an inherited complement deficiency.

## 2. Case

A 25-year-old African American woman initially presented to the hospital in 2014 with anosmia, blurry vision, and arthralgias. Subsequent serological evaluation led to a diagnosis of SLE (Table 1). She was started on disease modifying antirheumatic drug (DMARD) treatment with hydroxychloroquine 400 mg daily along with a tapering course of oral steroids. Her symptoms promptly improved. Her initial serological tests as shown in Table 1 revealed a number of abnormal lupus markers. She also had low serum C3 and low serum C4. Interestingly, her CBCAPs were negative. The low C3 and C4 at the time were attributed to complement consumption from active SLE. Nine months later, when her SLE was quiescent, her serological makers were remeasured (Table 2). As shown in Table 2, she continued to show several abnormal lupus serologies. Her serum C3 and C4 continued to remain low, and her CBCAPs remained negative. Approximately 18 months after her initial SLE diagnosis, the patient was hospitalized for fever, headache, and sinusitis. While initially thought to be a SLE flare, her blood cultures subsequently grew* Streptococcus pneumoniae* and she was treated for sepsis with antibiotics and clinically improved. The bacteremia was attributed to her sinusitis. Labs checked during this hospitalization again revealed a low serum C3 and C4 of 88 mg/dl and 5 mg/dl, respectively. In addition, a measured total hemolytic complement activity assay (CH50) was <10 *μ*/ml (reference range: 31–60 *μ*/ml). On further questioning, the patient acknowledged having many prior episodes of sinusitis and respiratory infections.

## 3. Discussion 

Deficiencies of early complement components are strongly associated with SLE or lupus like disease. This is hypothesized to occur for several reasons. Functionally, complement identifies, opsonizes, and disposes apoptotic cells and immune complexes formed between antibodies and foreign or self-antigens. The inability to clear these apoptotic cells can cause them to be a source of autoantigens and thereby drive autoantibody production [6–8]. This impaired clearance of immune complexes and other apoptotic “self” debris provides a logical explanation for complement deficiency in SLE [6, 9]. Another hypothesis that links complement deficiency with SLE is that the complement system is involved in immune tolerance. The early components of the complement pathway engage with the adaptive immune system to achieve tolerance against self-antigens. A complement deficiency that leads to a breach in self-tolerance can result in a lack of normal B cell tolerance, perpetuating autoantigens and immune complex formation [6, 10, 11].

Complement deficiency in humans can be inherited or acquired. Acquired complement deficiencies are quite common and can occur as a result of decreased synthesis, increased protein loss, or increased consumption. The liver produces several complement components, and low complements can be seen in individuals with advanced liver failure. Increased protein loss associated with nephritic syndrome or protein losing enteropathy can result in complement deficiencies [12]. Our patient did not have evidence of liver failure or protein loss. Inherited deficiencies in complement are complicated processes that are also closely linked to the development of SLE. In particular, the homozygous hereditary deficiency of each of the early components of the classical complement pathway is observed to be strongly associated with SLE [12, 13]. Deficiencies of components C1, C2, and C4 are inherited in an autosomal recessive manner and such deficiencies are the strongest genetic factors in susceptibility to developing SLE that have been characterized in humans [12]. The complexity in genetic control of levels of complement can be best exemplified by C4. There are two C4 genes in tandem, which encode C4A and C4B. C4A is important for solubilization of immune complexes and their clearance. C4 deficiency predisposes to the development of SLE [12]. In addition, partial deficiency of C4A or C4B is the most common inherited immune deficiency in humans, with a combined 30% in the normal Caucasian population [12, 14]. Yang et al. investigated the C4 genetic diversities in SLE in a study population of 216 female SLE patients, 17 male SLE patients, 362 first degree relatives, 389 unrelated healthy female controls, and 128 male controls. The study participants were all European Americans. Total gene copy-number (GCN) of C4 was analyzed and results showed that, in comparison to healthy controls, SLE patients had significant reductions in GCN of total C4. It was also noted that among SLE patients, 6.5% had a homozygous deficiency (i.e., 0 copies) of C4A and 26.4% had a heterozygous deficiency (i.e., one copy), compared to 1.3 and 18.2%, respectively, in healthy controls [6, 15]. The measured complement levels in this patient have shown consistently low levels of C4, while C3 levels have been more variable. It is thus not unreasonable to surmise that our patient may have a deficiency in C4. The CH50 test is a screening assay for activation of the classical complement pathway and reflects a reduction, absence, and/or inactivity of any component of the pathway [16]. The CH50 can be depressed in conditions that reflect decreased complement synthesis (inherited or acquired deficiency, malnutrition, and liver dysfunction) or increased consumption such as in bacterial infection, autoimmune disease, and organ transplant rejection. The CH50 level is zero if a complement component is absent, and the level is decreased if a classical pathway complement is decreased. Our patient's CH50 level of less than 10 *μ*/ml suggests absent complement and/or its components, possibly C4. In addition, deficiencies in early classical complement pathway components are also implicated in higher susceptibility to infections caused by encapsulated organisms including* Streptococcus pneumoniae, Haemophilus influenzae*, and* Neisseria meningitidis* [12, 17, 18].

This interesting case raises the possibility of our patient having an inherited complement deficiency as the etiology of her SLE. It also provides an explanation for her bacteremia and recurrent sinusitis. The rationale for this observation is that the patient has had continuously low levels of serum complement. Initially, it was thought that the low complement levels were due to consumption from active SLE. However, the patient's sera C3 and C4 have been low even when her SLE was inactive. In addition, the patient's measured CBCAPs were negative even when measured during an active lupus flare. An inherited complement deficiency can explain her chronically low complement levels and absent CH50 level. Her measured CBCAPs are most likely negative because there is not enough complement activation to produce a measurable quantity of complement split products. In addition, as observed by Calano et al., erythrocytes have a lifespan of 120 days and may bind and accumulate C4d throughout that period. Thus, EC4d levels would likely reflect the collective result of complement activation and SLE disease activity over a 120 day period [1]. This lends further credence to our postulate that the patient is not chronically producing CBCAPs.

Our observation is limited by certain considerations. First, a full evaluation of complement deficiency has not been done in this patient. Evaluating inherited complement deficiencies and analyzing complement GCN are complicated and not feasible in an outpatient clinical rheumatology practice. Another consideration is that the patient has an acquired complement deficiency rather than an inherited complement deficiency. Autoantibodies binding to complement proteins could lead to a state of an acquired deficiency and contribute to SLE pathogenesis similar to the way genetic deficiencies do [6]. The patient did have positive anti-C1q IgG antibodies. C1q antibodies are present in 2–8% of the healthy population but are present in 30–48% of SLE patients [17, 19]. C1q antibodies are associated with intense activation of the classical complement pathway resulting in low levels of C1q, C4, and C2 [17, 20]. Following the classical complement pathway activation, C1q remains attached to immune complexes and is therefore located at the site of inflammation. Proteases at the inflammatory site then degrade IgG and C1q, creating multiple proteolytic fragments of IgG and C1q [6]. While positive anti-C1q antibodies could explain low levels of C3 and C4 in this patient, it would still not explain why her CBCAPs would be negative. Anti-C1q antibodies would cause an intense consumption of complement, leading to an elevation in EC4d and BC4d, which did not occur in this patient.

## 4. Conclusion

CBCAPs have emerged as highly sensitive SLE indicators and are now routinely being measured in clinical practice along with traditional serological markers in diagnosing and assessing SLE activity. Further study is needed, but we believe that CBCAPs may play a novel role in predicting an inherited complement deficiency. We recommend clinicians consider this possibility when evaluating SLE patients with chronically low complement levels and negative CBCAPs.

## Figures and Tables

**Table 1:** July 09, 2014.

ANA	Result	Reference ranges
Immunofluorescence (IIF)	1 : 5120 (positive)/homogeneous	Negative (<1 : 80); positive (≥1 : 80)

Analyte	Result	Reference ranges

Anti-dsDNA (ELISA)	581 IU/mL (positive, confirmed by *Crithidia*)	≤301 (negative); > 301 (positive)
Anti-Smith (ELISA)	46 U/mL (positive)	<5 (negative); 5–10 (equivocal); > 10 (positive)
EC4d (FACS)	6 Net MFI (negative)	≤12 (negative); > 12–75 (positive); > 75 (strong positive)
BC4d (FACS)	34 Net MFI (negative)	≤48 (negative); > 48–200 (positive); > 200 (Strong Positive)
Anti-U1RNP IgG	13 U/ml (positive)	<5 negative; 5–10 equivocal; > 10 positive
Anti-RNP70 IgG	1 U/ml (negative)	<7 negative; 7–10 equivocal; > 10 positive
Anti-SS-A/Ro IgG	182 U/ml (positive)	<7 negative; 7–10 equivocal; > 10 positive
Anti-SS-B/La IgG	>320 U/ml (positive)	<7 negative; 7–10 equivocal; > 10 positive
Anti-C1q IgG	144 U/ml (positive)	<20 units (negative); ≥ 20 units (positive)
C3	53 mg/dl	90–180 mg/dl
C4	3 mg/dl	16–47 mg/dl

**Table 2:** April 1, 2015.

ANA	Result	Reference ranges
Immunofluorescence (IIF)	1 : 1280 (positive)/homogeneous	Negative (<1 : 80); positive (≥1 : 80)

Analyte	Result	Reference ranges

Anti-dsDNA (ELISA)	867 IU/mL (positive, confirmed by *Crithidia*)	≤301 (negative); > 301 (positive)
Anti-Smith (ELISA)	46 U/mL (positive)	<5 (negative); 5–10 (equivocal); > 10 (positive)
EC4d (FACS)	6 Net MFI (negative)	≤12 (negative); > 12–75 (positive); > 75 (strong positive)
BC4d (FACS)	43 Net MFI (negative)	≤48 (negative); > 48–200 (positive); > 200 (strong positive)
Anti-U1RNP IgG	14 U/ml (positive)	<5 (negative); 5–10 (equivocal); > 10 (positive)
Anti-RNP70 IgG	2 U/ml (negative)	<7 (negative); 7–10 (equivocal); > 10 (positive)
Anti-SS-A/Ro IgG	>240 U/ml (positive)	<7 (negative); 7–10 (equivocal); > 10 (positive)
Anti-SS-B/La lgg	>320 U/ml (positive)	<7 (negative); 7–10 (equivocal); > 10 (positive)
C3	69 mg/dl	90–180 mg/dl
C4	3 mg/dl	16–47 mg/dl

ELISA: enzyme-linked immunosorbent assay. FACS: fluorescence-activated cell sorting.
